# Extended-spectrum β-lactamase-encoding genes are spreading on a wide range of *Escherichia coli* plasmids existing prior to the use of third-generation cephalosporins

**DOI:** 10.1099/mgen.0.000203

**Published:** 2018-08-06

**Authors:** Catherine Branger, Alice Ledda, Typhaine Billard-Pomares, Benoît Doublet, Stéphanie Fouteau, Valérie Barbe, David Roche, Stéphane Cruveiller, Claudine Médigue, Miguel Castellanos, Dominique Decré, Laurence Drieux-Rouze, Olivier Clermont, Jérémy Glodt, Olivier Tenaillon, Axel Cloeckaert, Guillaume Arlet, Erick Denamur

**Affiliations:** ^1^​ IAME, UMR1137, INSERM, Université Paris Diderot, Sorbonne Paris Cité, UFR de Medecine, 16 Rue Henri Huchard, Paris 75018, France; ^2^​ Department of Infectious Disease Epidemiology, Imperial College, London, W2 1PG, UK; ^3^​ APHP, Service de Microbiologie Clinique, Hôpital Avicenne, 93000, Bobigny, France; ^4^​ ISP, INRA, Université François Rabelais de Tours, UMR 1282, 37380 Nouzilly, France; ^5^​ Laboratoire de Biologie Moléculaire pour l'Etude des Génomes, (LBioMEG), CEA, Genoscope, Institut de Biologie François-Jacob, 9100, Evry, France; ^6^​ UMR8030, CNRS, Laboratoire d'Analyses Bioinformatiques pour la Génomique et le Métabolisme, CEA, Institut de Génomique – Genoscope, Université Évry-Val-d'Essonne, 91000, Evry, France; ^7^​ IAME, UMR 1137, INSERM, Université Paris Diderot, Université Paris13, Sorbonne Paris Cité, 75018, Paris, France; ^8^​ CIMI, UMR 1135, INSERM, Université Pierre et Marie Curie Sorbonne Université, 75013, Paris, France; ^9^​ APHP, Hôpital de la Pitié-Salpêtrière Service de Bactériologie-Hygiène, 75015, Paris, France

**Keywords:** *Escherichia coli*, plasmid, extended-spectrum β-lactamase, CTX-M-15, gene-sharing network

## Abstract

To understand the evolutionary dynamics of extended-spectrum β-lactamase (ESBL)-encoding genes in *Escherichia coli*, we undertook a comparative genomic analysis of 116 whole plasmid sequences of human or animal origin isolated over a period spanning before and after the use of third-generation cephalosporins (3GCs) using a gene-sharing network approach. The plasmids included 82 conjugative, 22 mobilizable and 9 non-transferable plasmids and 3 P-like bacteriophages. ESBL-encoding genes were found on 64 conjugative, 6 mobilizable, 2 non-transferable plasmids and 2 P1-like bacteriophages, indicating that these last three types of mobile elements also play a role, albeit modest, in the diffusion of the ESBLs. The network analysis showed that the plasmids clustered according to their genome backbone type, but not by origin or period of isolation or by antibiotic-resistance type, including type of ESBL-encoding gene. There was no association between the type of plasmid and the phylogenetic history of the parental strains. Finer scale analysis of the more abundant clusters IncF and IncI1 showed that ESBL-encoding plasmids and plasmids isolated before the use of 3GCs had the same diversity and phylogenetic history, and that acquisition of ESBL-encoding genes had occurred during multiple independent events. Moreover, the *bla*_CTX-M-15_ gene, unlike other CTX-M genes, was inserted at a hot spot in a *bla*_TEM-1_-Tn*2* transposon. These findings showed that ESBL-encoding genes have arrived on wide range of pre-existing plasmids and that the successful spread of *bla*_CTX-M-15_ seems to be favoured by the presence of well-adapted IncF plasmids that carry a Tn*2-bla*_TEM-1_ transposon.

## Data Summary

One hundred and sixteen complete sequences of plasmids have been deposited in the European Nucleotide Archive (www.ebi.ac.uk/ena/) under the accession numbers: LT985213 to LT985387 (project PRJEB24625), FO818745, FQ482074, LO017736, LO017737 and LO017738. For individual plasmid accession numbers, please refer to Tables S1 and S2 (available with the online version of this article).

**Impact Statement**

Since the 2000s, an explosive spread of extended-spectrum β-lactamases (ESBLs), enzymes that hydrolyse and cause resistance to extended-spectrum cephalosporins, has been observed, impacting both human and animal health. Among ESBLs, CTX-M and especially CTX-M-15 type enzymes have taken over from the SHV and TEM type ESBLs, and *Escherichia coli* is now the major host. The large majority of these ESBLs are plasmid encoded. The data from our comparative whole plasmid sequence analysis give a picture of the evolutionary dynamics of acquisition of plasmid-borne ESBL-encoding genes in *E. coli*. The results indicate that ESBL-encoding genes have arrived multiple times on a wide range of pre-existing plasmids and that a highly dynamic pattern of mobility concerning different nested physical units represented by the ESBL-encoding gene, the multi-resistance region and the plasmid, multiplies the potential of ESBL spread. The results also highlight the importance of the well-adapted IncF plasmid associated with the Tn*2-bla*_TEM-1_ transposon in the successful spread of the CTX-M-15 ESBL in *E. coli.*

## Introduction

The emergence and spread of resistance to third-generation cephalosporins (3GCs), mediated mainly by extended-spectrum β-lactamases (ESBLs) [1], is an increasing health problem. An important component of this emergence is mediated by the spread of plasmid-borne ESBL-encoding genes [2]. The CTX-M family of ESBLs currently predominates worldwide and has taken over from the SHV and TEM type ESBLs that were predominant in the 1990s [3]. Among these, CTX-M-15 belonging to the CTX-M-1 group appears to be the most widespread, followed by CTX-M-14, another common variant of the CTX-M enzymes [4, 5]. In recent years, the prevalence of *Escherichia coli* that produce ESBLs has dramatically increased. Consequently, *E. coli* is now recognized as the major source of ESBLs [5, 6]. ESBL-producing *E. coli* are commonly isolated from community or hospital infections and from human faecal carriage, and are also increasingly detected in food-producing, companion and wildlife animals, as well as in the environment. As a consequence, these resistant *E. coli* can impact on both animal and human health [1, 5]. In addition to the selective pressure exerted by the use of 3GCs, other factors may contribute to the emergence and success of *E. coli* producing ESBLs and in particular those producing CTX-M-15 ESBLs: the mobile elements involved in the capture/mobilization of the ESBL-encoding gene, the genetic background of ESBL-carrying plasmids [7] and the host strain carrying the plasmid. For instance, *E. coli* clones of phylogenetic group B2 and sequence type (ST) 131; of phylogenetic group D, and ST315, ST393 and ST405; and of phylogroup F and ST648 have largely contributed to the dissemination of ESBL worldwide [8–11].

The objective of this work was to better understand the evolutionary dynamics of the acquisition of plasmid-borne ESBL-encoding genes in *E. coli*. Therefore, we first sequenced ESBL-encoding plasmids of diverse types in terms of incompatibility group, type of ESBL (CTX-M, SHV and TEM) and ecosystem (human or animal) originating from 73 ESBL *E. coli* strains. These strains were selected from well-characterized human and animal *E. coli* collections [12–18]. Second, we sequenced non-ESBL plasmids originating from 18 human and animal *E. coli* strains isolated before the introduction of the 3GCs in clinical therapy (ECOR collection and personal collections) [19, 20]. Third, we sequenced the plasmid content of an *E. coli* strain of the Murray collection isolated during the pre-antibiotic era [21, 22]. Using complete and circularized plasmid sequences, we performed comparative genomic analyses to determine the structure of the plasmid communities, as well as the phylogenetic relationships of the closely related plasmids. We also investigated the relationships between the plasmids and their main features, such as their origin, antibiotic gene content and size and the phylogenetic history of the host strain.

## Methods

### Bacterial strains and plasmids

To carry out our comparative plasmid analysis, we selected ESBL-encoding plasmids, and plasmids isolated before the use of the 3GCs originating from human and animal collections of *E. coli* strains. ESBL-encoding plasmids, previously obtained after transfer by conjugation in *E. coli* K-12 J53rif^r^ or, in absence of conjugation, by electroporation in *E. coli* K-12 DH10B from human and animal *E. coli* collections [12–18, 23], were selected according to diversity in terms of their incompatibility group determined by PCR based replicon typing (PBRT) [24] and of the type of ESBL-encoding gene they carried (Table S1). From the human ESBL-producing *E. coli* collections [12, 17, 18, 23], 63 plasmids were selected. Among them, 50 were typable by PBRT [IncF (*n*=18), IncA/C (*n*=12), IncI1 (*n*=8), IncN (*n*=5), IncL/M (*n*=4), IncK (*n*=2), IncY (*n*=1)] and 13 were non-typable. A total of 35 plasmids encoded a CTX-M-type ESBL [CTX-M-15 (*n*=13), CTX-M-14 (*n*=10), CTX-M-9 (*n*=2), CTX-M-3 (*n*=2), CTX-M-2 (*n*=2), CTX-M-1(*n*=6)], 12 a SHV-type ESBL [SHV-12 (*n*=7), SHV-2 (*n*=3), SHV-5(*n*=1), SHV-3(*n*=1)] and 16 a TEM-type ESBL [TEM-52 (*n*=5), TEM-24 (*n*=4), TEM-21 (*n*=2), TEM-3 (*n*=5)]. From the animal ESBL-producing *E. coli* collection [13–15], 10 plasmids, all typable by PBRT, were selected [IncF (*n*=1), IncI1 (*n*=7), IncHI1 (*n*=1) and IncHI2 (*n*=1)]. Nine plasmids encoded a CTX-M-type ESBL [CTX-M-1 (*n*=5), CTX-M-2 (*n*=3), CTX-M-15 (*n*=1)] and one plasmid encoded a TEM-52 ESBL.

Plasmids isolated before the use of the 3GCs came from: (i) 15 strains of the ECOR collection [19], which represent the diversity of the *E. coli* population, 6 were of human origin and 9 of animal origin; (ii) 3 strains of human origin isolated between 1958 and 1969 [20] (INRA, UR1282, personal collection); (iii) 1 strain of human origin from the Murray collection isolated before the use of antibiotics [21, 22] (Table S2). These strains, with the exception of two ECOR strains and the strain from the Murray collection, were resistant to at least one antibiotic. Resistant plasmids were transferred by conjugation in *E. coli* K-12 J53rif^r^ or, in the absence of conjugation, by electroporation in *E. coli* K-12 DH10B using one of the following antibiotics as selective agents, according to the resistance phenotype of the strain: ampicillin, streptomycin, tetracycline, sulfonamides. Eight of the resistant plasmids were typable by PBRT [IncF (*n*=4), IncI1 type (*n*=1), IncB/O type (*n*=2), IncX (*n*=1)] and eight were non-typable. PBRT of the antibiotic-sensitive ECOR strains showed that one had non-typable plasmids and the other a plasmid of IncF type. The Murray collection strain had a plasmid of IncF type.

### Parental strain chromosome phylotyping

The parental strains were assigned to one of the seven *E. coli* phylogenetic groups, A, B1, B2, C, D, E, F, or to *Escherichia* clade I using the quadruplex PCR-based method developed by Clermont *et al*. [25]. Multilocus sequence typing (MLST) was performed using the Institut Pasteur MLST (MLST IP) scheme based on the partial sequences of eight genes (*dinB*, *icdA*, *pabB*, *polB*, *putP*, *trpA*, *trpB*, *uidA*) as described previously [26] (http://bigsdb.web.pasteur.fr/). Phylogenetic analysis was performed with the concatenated sequences of the eight genes, using the maximum-likelihood method implemented in the PhyML program [27] with *Escherichia fergusonii* as the outgroup.

### Plasmid DNA sequencing and annotation

Plasmid DNA was purified from the *E. coli* K-12 recipient strains and from the three antibiotic-sensitive strains with the Macherey-Nagel nucleobond BAC100 kit and sequenced according to two strategies: transposition for small-sized plasmids (<30 kb) (template generation system II; Finnzymes) and high-density pyrosequencing on a 454 GSFlx instrument with titanium chemistry (Roche) for large-sized plasmids (>30 kb). The reads generated, of a mean length of 350 bp (40× coverage), were assembled *de novo* by the Newbler assembler [28] into contigs and produced circularized sequences. Combinational PCRs and Sanger sequencing were used to fill in gaps.

An automatic annotation was undertaken using the MicroScope platform (www.genoscope.cns.fr/agc/microscope) [29]. Genomic object annotations were subsequently validated by a manual expert annotation. All the data generated during the annotation processes were integrated into the Prokaryotic Genomic DataBase (PkGDB) browsable via the MicroScope platform GUI. Annotation of insertion sequences was performed using the IS Finder resource (https://isfinder.biotoul.fr/) [30].

### Classification of the plasmids

The plasmids were classified according to their mobility characteristics as described elsewhere [31]. Conjugative plasmids having a set of genes encoding a mating pair formation (MPF) and a relaxase were called MPF plasmids. Plasmids having a relaxase gene but no MPF genes were called MOB (mobilizable) plasmids. Plasmids having no MPF genes and no relaxase gene were called RelN (relaxase negative) plasmids (non-transferable plasmids). MPF plasmids were classified further according to their incompatibility (Inc) group [24], and the MOB and RelN plasmids according to the type of their replication system: MOB_RNA_ and RelN_RNA_ for plasmids with a RNAII/RNAI replication system similar to that of the colE1 plasmid [32, 33], and MOB_rep_ and RelN_rep_ for plasmids with a replication protein system. Plasmid MLST (pMLST) was performed on complete sequences of IncF, IncI1 and IncN by submitting the amplicon sequence to the Plasmid MLST website (http://pubmlst.org/plasmid/) [34].

### Statistical analysis

To describe associations between variables, a factorial analysis of correspondence (FAC) was conducted. FAC uses a covariance matrix based on χ^2^ distances. R software [35] (http://CRAN.R-project.org) was used for FAC with a two-way table. The table had 116 rows, corresponding to the 116 plasmids studied and 14 columns corresponding to the 14 variables: plasmid type (MPF, MOB, RelN and phage), type of ESBL-encoding gene (CTX-M, SHV, TEM), plasmids with non-ESBL resistance genes, plasmids with no resistance genes, size of the plasmids (0–30 kb, 30–100 kb, 100–>200 kb) and period of isolation (before or after the use of 3GCs). For each column, each plasmid was coded as a binary code: present=1, absent=0.

### Comparative genomic analyses of the sequenced plasmids

To reconstruct the evolutionary history of the plasmids by identifying those having the most similar gene content, comparative genomic analyses were performed. For each plasmid, the annotated gene sequences and the whole genome (scaffold) sequence were downloaded from the PkGDB database (Prokaryotic Genomic DataBase) [29]. The whole genomes were concatenated to create a blast database [36, 37]. Each gene present in the annotated genomes was then blast analysed on the whole genome database with a strict *e*-value (0.0001). We thus obtained a matrix of the number of genes shared between each pair of plasmids. We used the Jaccard distance (JD) to transform the matrix of shared genes between any two plasmids into a matrix of distance between the plasmids. The JD between any two plasmids is defined as the percentage of non-shared genes between the two plasmids, being 0 when two plasmids have the exact same gene content and 1 when two plasmids do not share any genes. The distance between any two plasmids can assume any value between 0 and 1, depending on the number of genes they share and the total number of genes present in the two plasmids. As many of the plasmids have few or no genes in common, a standard gene clustering is not suited for our plasmid database. We thus developed a phylogenetic approach inspired from network theory [38] to cluster the plasmids. Using the package Igraph [39] from the R software [35], we explored how the plasmids are attached at different thresholds in the JD from its minimum distance (JD=0) to its maximum distance (JD=1). In particular, we focused on the behaviour of two quantities: the size of the biggest cluster of connected plasmids [40] and the number of clusters, which were computed using in-house R scripts. The network was drawn using the function plot.igraph. Each node of the network stands for a plasmid and a link between two nodes is drawn. The clusters were isolated and a subset of plasmids having the same set of shared genes were manually isolated in each cluster [41]. For each subset of plasmids, the set of shared genes were retrieved and concatenated using Perl scripts. The concatenated sets of genes were then aligned using the program Mauve [42] and a tree was built using the program PhyML [27] under the evolution model GTR (general time reversible).

## Results

### Sequencing of the plasmid collection

Sequencing of the plasmid content of the 89 recipient strains, which exhibited at least one plasmid-borne antibiotic-resistance gene, showed that 14 of them contained two to four different types of plasmids. Of these 14 recipient strains, 11 were transconjugants (21.5 %) and contained 26 plasmids (mean=2.36), and 3 were electroporants (7.8 %) and contained 6 plasmids (mean=2). Direct plasmidome sequencing of the three sensitive strains showed that the strains contained two to four different types of plasmids (mean=3) (Table S2). Therefore, a total of 116 plasmids was further studied, including 74 ESBL-resistance plasmids, 17 non-ESBL-resistance plasmids and 25 plasmids without any resistance genes. Among these, 87 and 29 originated from strains isolated before and after the use of 3GCs, respectively, and 91 and 25 originated from human and animal strains, respectively.

### Classification of the plasmids

First, we classified the plasmids as described in Methods. A total of 82 of the 116 plasmids were MPF plasmids, which included 26 IncF, 17 Inc I1, 13 IncA/C, 5 IncN, 4 IncL/M, 2 IncK, 2 IncB/O, 2 IncHI, 3 IncX1, 2 IncX4, 1 IncX2, 1 IncN2, 1 IncY, 1 IncX-like, 1 IncN-like, 1 IncNA (NA, non-attributed); 22 plasmids were MOB plasmids that included 17 MOB_RNA_ plasmids and 5 MOB_rep_ plasmids; and 9 plasmids were RelN plasmids that included 8 RelN_RNA_ plasmids and 1 RelN_rep_ plasmid of IncR type (Table 1). In addition, three temperate bacteriophages that replicate like plasmids were evidenced. Two displayed a high level of identity to the P1-like bacteriophage genomes [43, 44] and one to the P2-like bacteriophage genomes [45] (Table 1).

**Table 1.** Distribution of the plasmids studied according the type of mobilization and replication/control system, the presence of resistance genes and the type of ESBL-encoding gene carried

Plasmid type	No. of plasmids	No. of plasmids encoding at least one ESBL																	No. of non-ESBL-encoding plasmids with resistance genes	No. of plasmids without resistance genes
		CTX-M							TEM					SHV						
		-1	-2	-3	-9	-14	-15	Total	-3	-21	-24	-52	Total	-2	-3	-5	-12	Total		
MPF plasmids*																				
IncF	26		1		1	6	7	**15**						2	1		2	**5**	**5†**	**1†**
IncI1	17	6	1				2	**9**				3	**3**	1				**1**	**2†**	**2‡**
IncA/C	13		1			1	1	**3**	3	2	3	1	**9**				1	**1**		
IncL/M	4			2		1		**3**	1				**1**							
IncN	5	4					1	**5**												
IncX group	6						2	**2**				1	**1**						**2†**	**1†**
IncX1	3						1					1							**1†**	
IncX2	1																		**1†**	
IncX4	2						1													**1†**
IncHI	2		2					**2**												
IncK	2					2		**2**												
IncB/O	2																		**1†**	**1†**
IncY	1										1		**1**							
IncN2	1															1		**1**		
IncX-like	1																			**1‡**
IncN-like	1																		**1†**	
IncNA	1																			**1†**
**MOB plasmids§**																				
MOB_rep_	5						1	**1**											**1†**	**3‡**
MOB_repB1_	4						1													**3‡**
MOB_repB2_	1																		**1†**	
MOB_RNA_	17								1				**1**				4	**4**	**1‡, 1†**	**6‡, 4†**
**RelN plasmids||**																				
IncR	1											1								
RelN_RNA_	8					1		**1**											**4†**	**3†**
**Phage**																				
P1-Like	2				1			**1**									1	**1**		
P2-like	1																			**1†**
**Total**		10	5	2	2	11	14	**44**	5	2	4	6	**17**	3	1	1	8	**13**	**18**	**24**

*MPF conjugative plasmids [31].

†Isolated before the use of 3GCs.

‡Isolated after the use of 3GCs.

§MOB_rep_, plasmids with a replication protein system; MOB_RNA_, plasmids with a RNAII/RNAI replication/control system.

||RelN_RNA_, plasmids with a RNAII/RNAI replication/control system.

The sequencing results showed that the PBRT non-typable ESBL-encoding plasmid selected for the study [24] had a replicon type that was not included in the panel used at the time of the typing or not yet available or that the plasmids had mismatches in the primer sequences used for typing.

Besides the IncF plasmids, which frequently display several replicons (FII with or without FIA/FIB/FIC) [46], some of the plasmids carried supplementary replicons (Tables S1 and S2). The two replication protein genes, *repA* and *repC*, of an IncQ-1 plasmid [47] were found inserted between two IS*26* elements on two IncFII-FIB plasmids, one IncHI1 plasmid and one P1-like bacteriophage. A complete core genome of an IncR plasmid [48] and a partial core genome of an IncN2 plasmid [49] were found on two IncA/C plasmids. A FIB replicon was found inserted between two IS*629* elements on a MOB_RNA_ plasmid. As noted by Osborn *et al*. [50], the existence of mosaic replicons causes additional complications for the classification of bacterial plasmids and for attempts to assess the evolutionary relationships between plasmids and their replicons [50].

### Relationships between the plasmids and their main characteristics

**Fig. 1.**
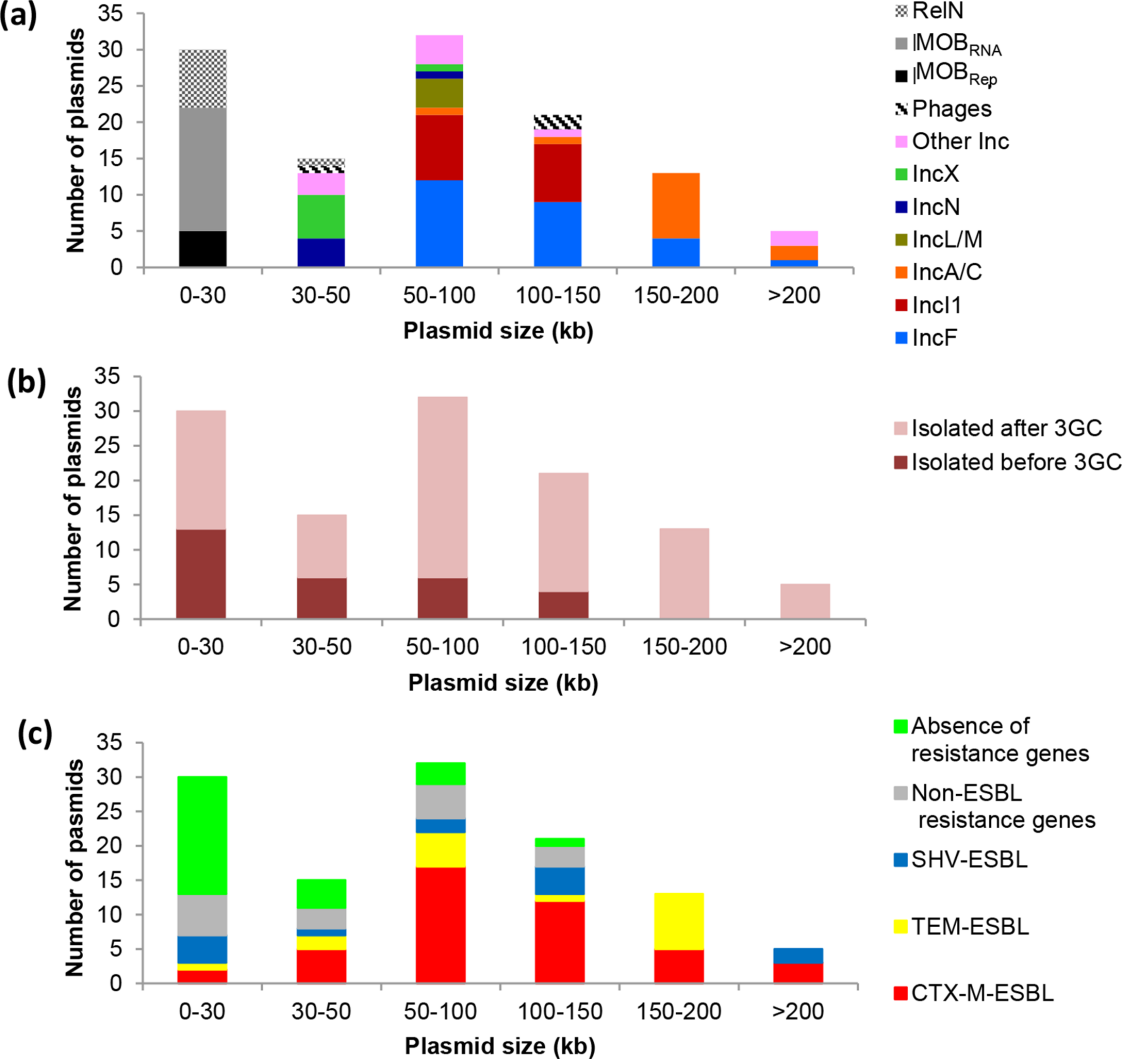
Distribution of the characteristics of the plasmids according to their size (kb). (a) Type of plasmids: MPF plasmids [31] indicated by their incompatibility group (Inc), MOB plasmids indicated by their replication system (MOB_rep_ for replication protein system and MOB_RNA_ for a RNAI/RNAII replication/control system) and RelN plasmids (non-transferable). (b) Isolation period of the plasmids: before or after the use of 3GCs. (c) Resistance type: plasmids with at least one ESBL-encoding gene, plasmids with non-ESBL resistance genes and plasmids with no resistance genes.

We next studied various characteristics of the plasmids, i.e. their size and the presence or absence of antibiotic-resistance genes, as well as the period of isolation of the strain (after or before the use of 3GCs). First, as observed elsewhere [31], all the MOB and the RelN_RNA_ plasmids had a small size lower than 25 kb with a mean of 9.4 kb (2.9–23.5 kb), and all the MPF plasmids, the bacteriophage and the RelN-IncR plasmid had a size higher than 30 kb with a mean size of 104 kb (34–239 kb). Most of the smallest MPF plasmids (30–55 kb) were the IncN and IncX plasmids, and most of the biggest plasmids (>150 kb) were the IncA/C plasmids (Fig. 1a). Secondly, of the 87 plasmids isolated after the use of the 3GCs, we identified 67 MPF plasmids (20 IncF, 15 IncI1, 13 IncA/C, 4 IncL/M, 5 IncN, 2 IncK, 2 IncX1, 1 IncX4, 2 IncHI, 1 IncY, 1 IncN2 and 1 IncN-like plasmid), 4 MOB_rep_ plasmids, 12 MOB_RNA_ plasmids, 1 RelN-IncR plasmid, 1 RelN_RNA_ plasmid and 2 P1-like bacteriophages. CTX-M-type genes were carried by 40 MPF plasmids, 1 MOB_rep_ plasmid, the RelN_RNA_ plasmid and 1 P1-like bacteriophage. ESBL-TEM-type genes were carried by 16 MPF plasmids, the RelN-IncR plasmid and 1 MOB_RNA_ plasmid. Lastly, ESBL-SHV-type genes were carried by eight MPF plasmids, four MOB_RNA_ plasmids and one P1-like bacteriophage (Fig. 1b, c, Tables 1 and S1). The 29 plasmids isolated before the use of the 3GCs included 15 MPF plasmids (six IncF, two IncI1, two IncB/O, one IncX1, one IncX2, one IncX4, one IncX-like and one IncNA), 1 MOB_rep_ plasmid, 5 MOB_RNA_ plasmids, 7 RelN_RNA_ plasmids and 1 P2-like bacteriophage. Resistance genes were found on 11 MPF plasmids, on 1 MOB_RNA_ plasmid, on the MOB_rep_ and on 4 RelN_RNA_ plasmids (Fig. 1b, c, Tables 1 and S2).

**Fig. 2.**
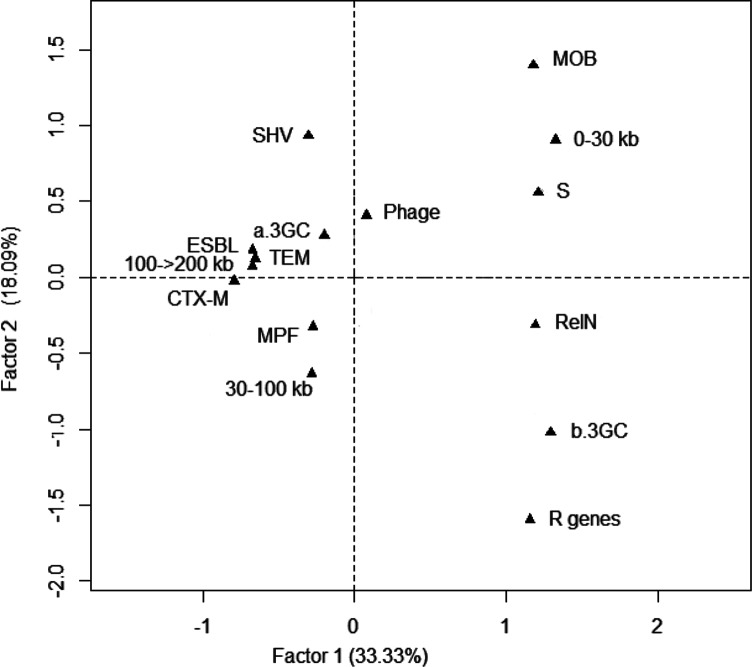
Graphical representation of the results of the FAC carried out with whole data from the 116 *E. coli* plasmids. Projections of the variables on the F1/F2 plane: type of plasmids (MPF, MOB, RelN and phage), size of the plasmids (0–30, 30–100, 100–>200 kb), resistance type [plasmids with at least one ESBL-encoding gene (ESBL), plasmids with non-ESBL resistance genes (R genes), plasmids with no resistance genes (S, sensitive)], type of ESBL-encoding gene (TEM, SHV, CTX-M) and period of isolation [before the use of the 3GCs (b.3GC) or after the use of the 3GCs (a.3GC)]. The percentage of the total variance represented by each factor is indicated.

To assess the global relationships between the plasmid type (MPF, MOB, RelN or phage), size, period of isolation, type of resistance and type of ESBL-encoding gene carried by the plasmids, a FAC was conducted with the 116 plasmids as individuals and the 16 characteristics as qualitative variables. Projections of the variables on the plane F1/F2, which accounted for 52.4 % of the total variance, showed that the variables plasmid isolated before (b.3GC) or after (a.3GC) the 3GCs were clearly distinguished by the first factor and that there was an association with the variables type of plasmid, type of resistance and size. The variable b.3GC was projected on the positive value of F1 with the variables MOB, RelN, size of 0–30 kb, plasmid with no resistance gene or plasmid with non-ESBL resistance genes, whereas the a.3GC variable was projected on the negative value of F1 with the variables MPF, sizes of more than 30 kb and presence of an ESBL-encoding gene. On the positive value of F1, the FAC showed a clear association between the variables MOB plasmid and plasmid with no resistance gene both projected on the positive value of F2, and an association between the variables RelN plasmid and plasmid with non-ESBL resistance gene both projected on the negative value of F2 (Fig. 2).

### Plasmid cluster determination

**Fig. 3.**
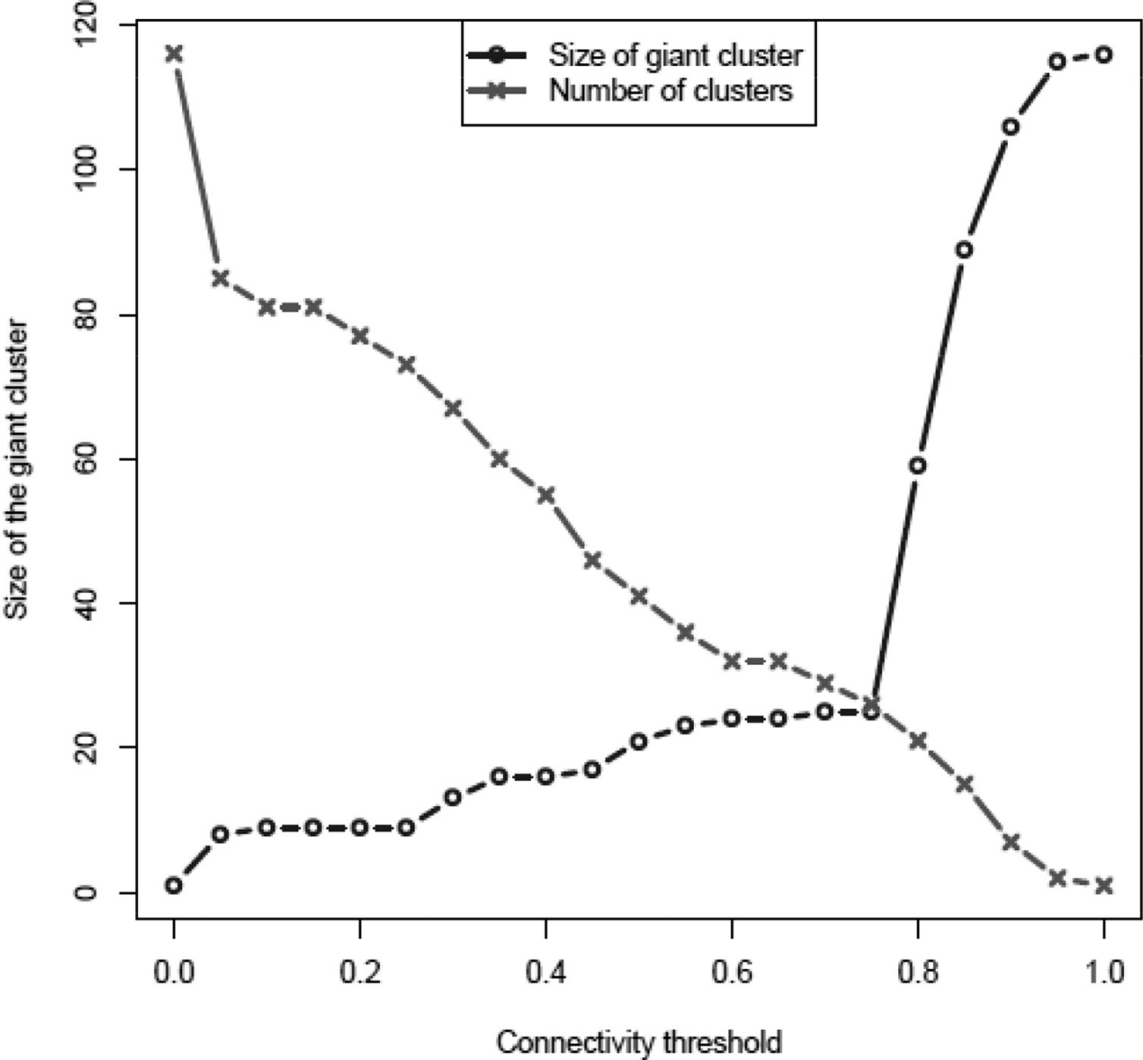
Dimension of the giant cluster as a function of the threshold in minimum number of genes by two plasmids. The line with crosses represents the number of clusters of connected plasmids at different distances between the plasmids, while the line with circles represents the size of the biggest cluster of plasmids (giant component) at different distances between the plasmids. Note the intersection of the lines at the connectivity threshold (0.75).

**Fig. 4.**
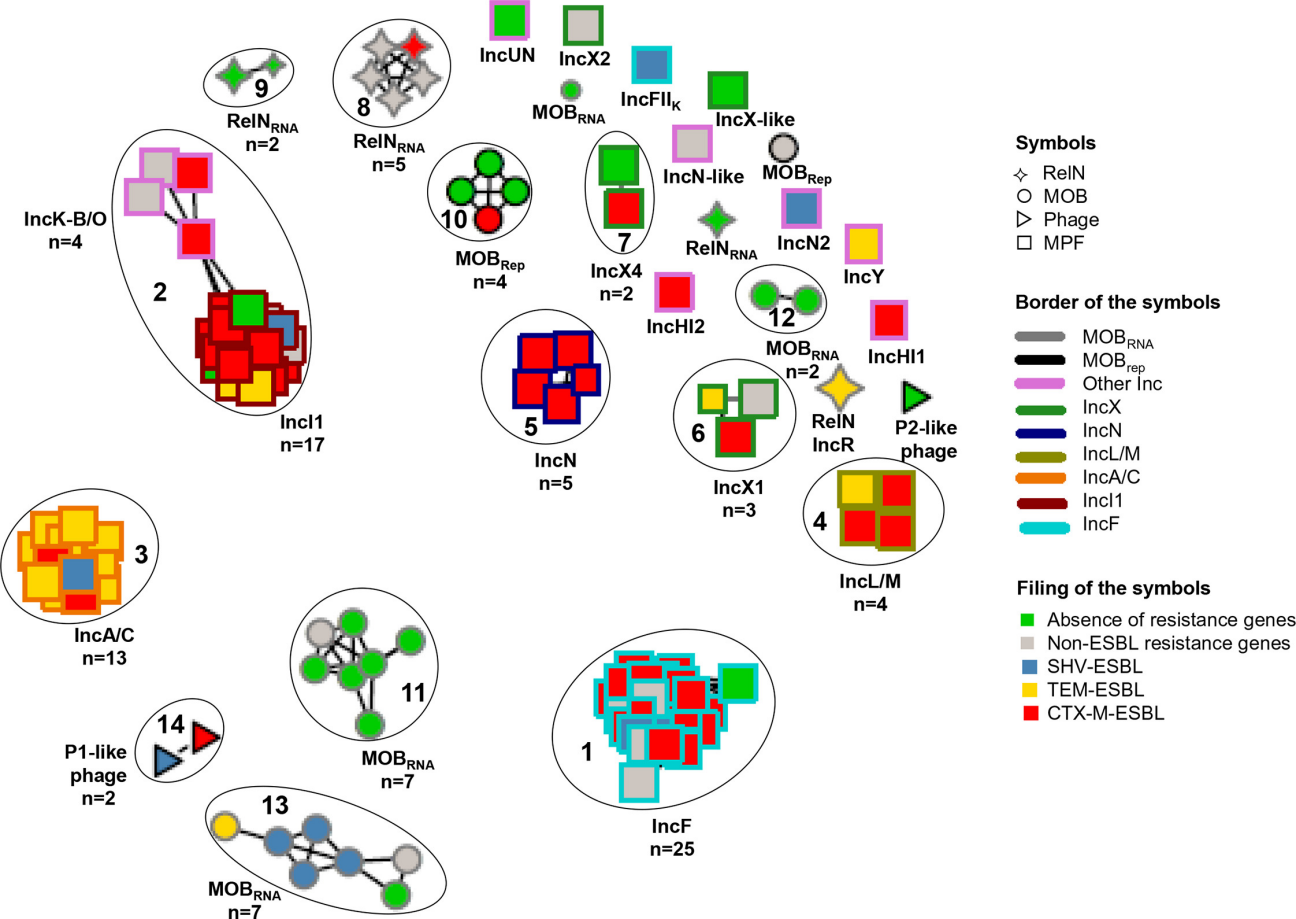
Graph of the plasmid network at a JD of 0.75 (percentage of non-shared genes between two plasmids). Symbols indicate the type of plasmid or phage: MPF, MOB and RelN. Colour codes of the borders of the symbols indicate the type of incompatibility group (Inc) for the MPF plasmids and the type of replication system for the MOB plasmids. MOB_RNA_ is for plasmids with an RNAII/RNAI replication system and MOB_rep_ for plasmids with a replication protein system. The fill colour of the symbols indicates the plasmid's type of resistance: red, blue and yellow are for plasmids carrying at least one ESBL-encoding gene. Clusters of at least two plasmids are surrounded in black and numbered.

Plasmid clusters were obtained as described in Methods using a phylogenetic approach inspired from network theory [38]. After computing the JD between any pair of plasmids in terms of the plasmids’ gene content, we looked for an optimal data driven distance threshold allowing definition of clusters composed of related plasmids. To this end, we focused on two quantities: the size of the biggest cluster of connected plasmids (called giant component) [40] and the number of clusters as a function of the connectivity threshold. The size of the biggest cluster (Fig. 3) has clearly two regimes: one for low connectivity thresholds (up to 0.75) where the size is almost stable, and one for high thresholds, where the size of the biggest cluster grows quickly until all the plasmids are linked in a single connected component. The sudden transition between these two regimes is related to a well-studied phenomenon in network theory called percolation [38, 40]. In the present analysis, the percolation transition can be explained as the point where the plasmids’ phylogenetic structure is shrouded by horizontal gene transfer (HGT). Below this percolation threshold, we assumed that plasmids were clustered by descendants and that the noise due to HGT was minimal. Thus, the plasmid network was drawn with links between two nodes (plasmids) when their distance was at most 0.75. Using this approach, the graph obtained showed that 102 plasmids were divided into 14 clusters of at least two plasmids (the clusters are surrounded in black and numbered in Fig. 4). A further 14 plasmids were not linked to any other plasmid (singleton plasmids).

### Cluster analysis

We first correlated the clusters obtained with the classification made previously on mobility type and of replication/control type systems. Among the 14 clusters, 7 contained MPF plasmids (clusters 1 to 7), 2 contained RelN_RNA_ plasmids (clusters 8 and 9), 1 contained MOB_rep_ plasmids (cluster 10), 3 contained MOB_RNA_ plasmids (cluster 11 to 13) and 1 contained phages (cluster 14) (Fig. 4). We then explored further the plasmid content of the various clusters.

### MPF clusters

In each of the seven MPF clusters, plasmids were of the same incompatibility group or complex. Cluster 1 contained 25 of the 26 IncF plasmids. Only an IncFII_k_ plasmid (RCS36) encoding a SHV-3 ESBL (Table S1) was found as a singleton, clearly showing that this plasmid type, described in *Klebsiella pneumoniae* strains [46], differed from the other IncFII plasmids. Cluster 2 contained the 17 IncI1 plasmids, the 2 IncK plasmids and the 2 IncB/O plasmids, which is consistent since all these plasmids belong to the I-complex [51]. Cluster 3 contained the 13 IncA/C plasmids, and cluster 4 the 4 IncL/M plasmids. Cluster 5 contained the five IncN plasmids excluding the IncN2 plasmid and the IncN-like plasmid found as singletons, showing that these latter plasmids have a low degree of sequence similarity with the IncN plasmids. Of the seven IncX plasmids, cluster 6 contained the three IncX1 plasmids and cluster 7 the two IncX4 plasmids, excluding the IncX2 and IncX-like plasmids found as singletons (Tables S1 and S2). The IncX-type plasmids, which are known to be diverse [52, 53], are clearly clustered according to their IncX-subgroup, stressing the low degree of sequence similarity between the IncX sub-types.

### RelN_RNA_ clusters

Of the eight RelN_RNA_ plasmids, five were found in cluster 8, two in cluster 9 and one was a singleton, corresponding to three different families of plasmids. Indeed, in each cluster the plasmid backbones were closely related, while between the clusters the plasmid backbones had no sequence similarity.

### MOB_rep_ cluster

Of the five MOB_rep_ plasmids, four were found in cluster 10 and one was a singleton. Plasmids of cluster 10 were closely related and had no sequence similarity with the singleton plasmid, showing two different plasmid families that we named MOB_repB1_ for the plasmids of cluster 10 and MOB_repB2_ for the singleton (Tables S1 and S2).

### MOB_RNA_ clusters

The 17 MOB_RNA_ plasmids were distributed in three clusters (clusters 11, 12 and 13) and only 1 plasmid was a singleton. To better characterize these three MOB_RNA_ plasmid clusters, we looked at their type of mobilization system and typed them *in silico* by the plasmid relaxase gene typing (PRaseT) developed by Compain *et al.* [54]. Cluster 11 contained seven MOB_RNA_ plasmids. Six of them had a backbone similar to the colE1 backbone [32] that includes a mobilization system, *mbeABCDE* [55], of relaxase gene type (RGT) P5-1 as colE1. The last plasmid did not share these characteristics and, therefore, belonged to a different family of plasmids (see below). An HGT containing a colicin E1 carried by this last plasmid and three plasmids of the cluster blurred the phylogenetic signal. Cluster 12 contained two plasmids that had a mobilization system that diverged from the one of colE1 and were not typed by PRaseT. Thus, they were considered as belonging to a different plasmid family. Cluster 13 contained seven plasmids that according to their mobilization system belonged to three different families. In this cluster, the effect of HGT hindered the phylogenetic signal. Indeed, a large resistance module carrying the *bla*_SHV-12_ gene acquired by four plasmids belonging to each of the three families linked the plasmids together and thereby plasmids of their respective families (data not shown). In this cluster, three plasmids had a mobilization system *mobABCD* of RGT P5-2 as pTPqnrS-1a, two plasmids had a mobilization system *mobBC* of RGT C11 as ColEST258 and the last two plasmids had a unique small relaxase gene, *mob,* of RGT P5-3 as pHUSEC41-4. These last two plasmids had the same backbone as the plasmid linked by HGT to the colE1-type plasmids of cluster 11 and the MOB_RNA_ singleton plasmid.

### Phage cluster

The two P1-like bacteriophages were found in cluster 14, while the P2-like bacteriophage was found as a singleton showing that these two types of phage had no sequence similarity.

### Singletons

In addition to the singleton plasmids previously cited, four other MPF plasmids were found as singletons: the two IncHI plasmids, one of subgroup IncHI1 and the other of subgroup IncHI2, stressing the low degree of sequence similarity between the two IncHI subgroup [56], the only plasmid of IncY group, and a plasmid that showed no sequence similarity with any of known MPF plasmid. The last singleton was a RelN_rep_ plasmid of IncR group [48] (Table 1).

### Relationship between the types of plasmids and the phylogeny of the parental strains

**Fig. 5.**
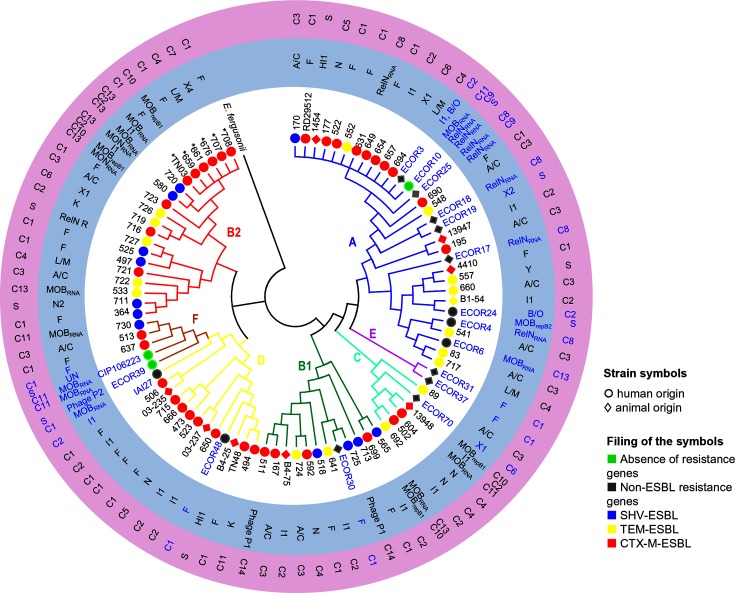
Phylogeny of the *E. coli* host strains. Phylogenetic tree reconstructed from the alignment of the concatenated sequences of eight genes of the MLST IP scheme using PhyML under the GTR model, with *E. fergusonii* as the outgroup. The phylogroups are indicated with the colours blue (A), green (B1), red (B2), turquoise (C), yellow (D), purple (E) and brown (F). First circle (no background): strain ID in blue for the strains isolated before the use of 3GCs and in black for the strains isolated after the use of 3GCs. * indicates strains of ST43 (ST131 Achtman MLST scheme); symbols indicate strain origin, fill colour of the symbols indicates the type of resistance (red, blue and yellow are for strains carrying at least one ESBL-encoding gene). Second circle (blue background): corresponding plasmids studied for each strain, MPF plasmids are indicated by their Inc group, and MOB plasmids and RelN plasmids by the type of replication system. Third circle (purple background): cluster number (C1 to C13) to which the plasmids belong, as defined in Fig. 4, and S (singleton) for plasmids not linked to a cluster.

We explored the clonal diversity, using the MLST IP [26], of the available parental strains of the plasmids studied (72/73 of the ESBL-producing strains and 17/19 of the strains isolated before the use of the 3GCs) in relation to the type of plasmid. Parental strains were distributed into 6 of the 7 lineages belonging to *E. coli sensu stricto* [25] (phylogroups A, B1, B2, D, C and F for the ESBL-producing strains and phylogroups A, B1, D, C, E and F for the strains isolated before the use of 3GCs) and showed a high diversity (Fig. 5). In each of the phylogroups, the diversity of the plasmids in terms of genome backbone and clusters and the diversity of the type of resistances was high underlying that, in each cluster of plasmids, the plasmids originated from *E. coli* strains of various backgrounds.

### Phylogeny of the IncF plasmids of cluster 1 and IncI1 plasmids of cluster 2

**Fig. 6.**
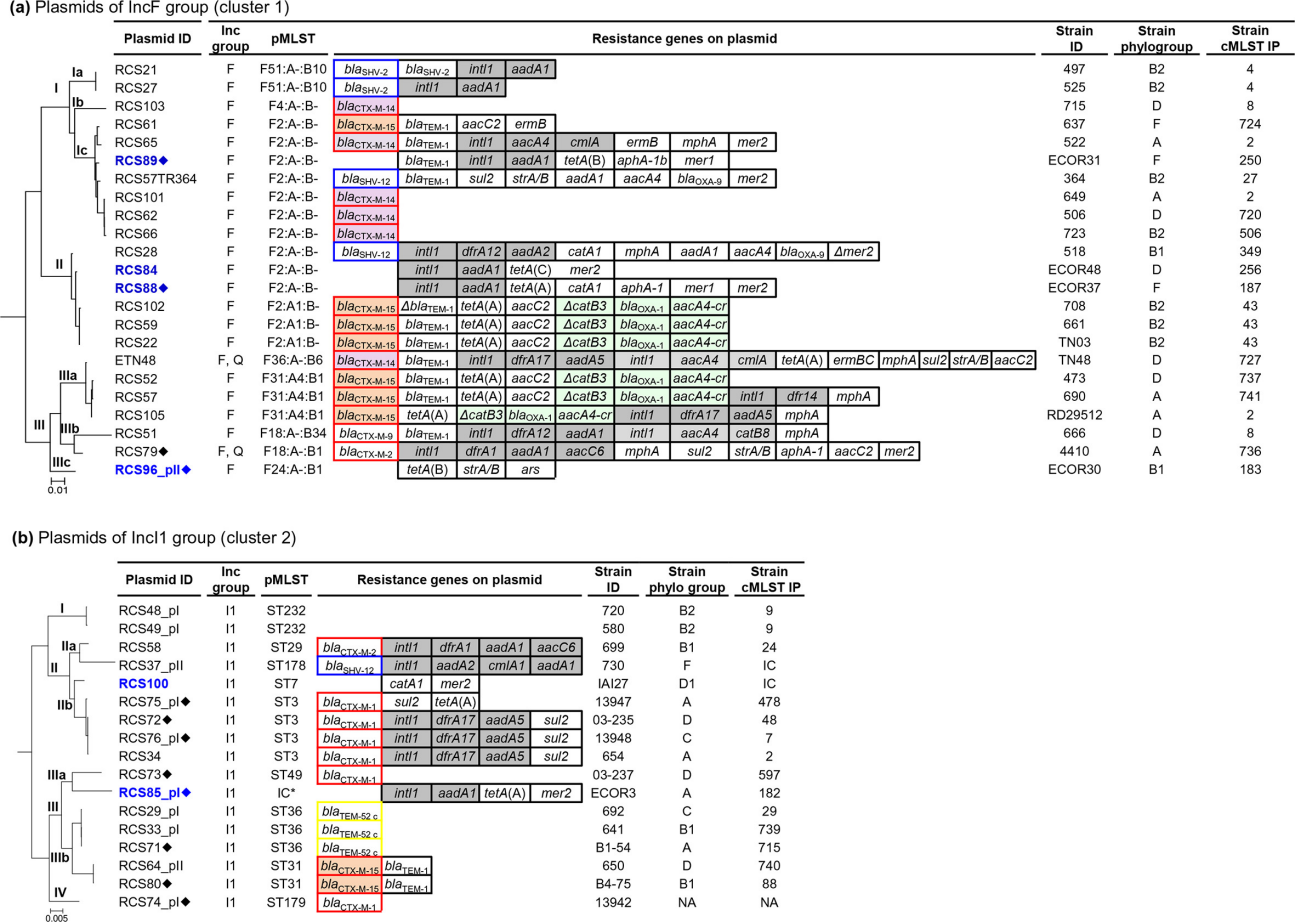
Phylogenetic trees of (a) 23 IncFII plasmids and (b) 17 IncI1 plasmids reconstructed as described in Methods. pMSLT, resistance genes on the plasmids, parental strain phylogroup and Institut Pasteur scheme chromosomal MLST (cMLST IP) are indicated to the right of the tree. Plasmid IDs are in blue for the strains isolated before the use of 3GCs and in black for the strains isolated after the use of 3GCs. ♦, plasmids isolated from animal strains. ESBL-encoding genes are framed in red for CTX-M-type genes, yellow for TEM-type genes and in blue for SHV-type genes. *bla*_CTX-M-14_ genes are shaded in violet and *bla*_CTX-M-15_ genes in orange. Integrons and their gene cassettes are shaded in grey. The IS*26* transposition *ca*t-*bla*_OXA-1-_*aacA4-cr* cassette array is shaded in turquoise. NA, Non-available; IC, incomplete ST. Bar, the approximate distance of substitution of nucleotides per site.

To explore more thoroughly the results presented above on a global scale, we analysed at a finer scale the evolutionary history of the plasmids for the two clusters containing the most plasmids, i.e. the MPF clusters 1 and 2 (Fig. 6). Complete data of the other clusters will be presented elsewhere. We reconstructed the phylogenetic trees using for each cluster of plasmids a pool of shared genes as described in Methods. As in each group of plasmids none of the plasmids shared a common resistance gene, the resistance genes did not influence the evolutionary history of the plasmids.

### IncF plasmids of cluster 1

The core genomes of IncFII plasmids have a mosaic structure [57] and have been shown to have an extensive diversity [58]. As the more genes we use the more reliable is the tree, we built a tree with 25 genes (the replication gene *repA1* and 24 genes of the *tra* operon) shared by 23 of the 25 plasmids of the cluster. The two excluded plasmids were RCS93_pI, originating from a strain of the Murray collection [21, 22], in which all but three of the *tra* operon genes were missing, and RCS70 that belonged to group C of the IncF/MOBF_12_ plasmids [59], while all the other IncFII plasmids of the cluster belonged to group A.

The phylogenetic tree obtained showed that the 23 IncF plasmids were distributed in three main branches, I to III, divided in sub-branches (Fig. 6a). Distribution of the plasmids in the branches was correlated with the plasmid STs (pSTs) attributed to the plasmids by pMLST [34]. However, pMLST lacked the sensitivity to discriminate the plasmids as pST F2 was attributed to 13 out of 23 plasmids that were distributed in two very divergent branches of the phylogenetic tree (7 in branch I and 6 in branch II). The four plasmids isolated before the use of the 3GCs, all from ECOR strains (three from animals and one from humans), were found distributed in the three branches of the phylogenetic tree along with ESBL-encoding plasmids, showing that ESBL-encoding plasmids have the same diversity and phylogenetic history as plasmids isolated before the use of the 3GCs (Fig. 6a).

We looked at the type of ESBL found on plasmids of the different branches and sub-branches. The seven *bla*_CTX-M-15_ plasmids were distributed in the three branches of the tree, the six *bla*_CTX-M-14_ plasmids were distributed in two branches (branches I and III), and the two *bla*_SHV-12_ plasmids were distributed in two branches of the tree (branches I and II). Only the two *bla*_SHV-2_ plasmids closely clustered in sub-branch Ia. Furthermore, closely related plasmids carried different ESBL-encoding genes, such as plasmids of sub-branch Ic that carried either a *bla*_CTX-M-15_, a *bla*_CTX-M-14_ or a *bla*_SHV-12_ gene, plasmids of branch II that carried either a *bla*_CTX-M-15_ or a *bla*_SHV-12_ gene, and plasmids of sub-branch IIIa that carried either a *bla*_CTX-M-15_ or a *bla*_CTX-M-14_ gene.

With the exception of four *bla*_CTX-M-14_ genes, all the other ESBL-encoding genes were found associated with various resistance modules on multi-resistance regions (MRRs) (Fig. 6a). However, five of the *bla*_CTX-M-15_ genes were on similar MRRs containing the same three resistance modules [a *tetA(A)* module, an *aacC2* module and an IS*26*-mediated cassette array (IS*26-aacA4-cr-bla*_OXA-1_-*ca*t*B3Δ*- IS*26*)] [60–62]. These particular *bla*_CTX-M-15_ MRRs were carried by three closely related plasmids of branch II (RCS22, RCS59 and RCS102) and two plasmids (RCS52 and RCS57) of branch IIIa showing movement of the association resistance module-*bla*_CTX-M-15_ gene between distantly related plasmids.

As observed in Figs 5 and 6(a), the background of the parental strains inferred by the phylogenetic group and the MLST IP was very diverse: the 23 strains were represented by five of the seven phylogroups that included 18 STs. Similar plasmids isolated from *E. coli* strains of the same phylogenetic background were demonstrated twice: in branch Ia, the two *bla*_SHV-2_ plasmids of pST F51 : A-B10 originating from B2-ST4 strains; and in branch II, the three *bla*_CTX-M-15_ plasmids of pST F2 : A1 : B- originating from B2-ST43-H30 strains (ST131, Achtman MLST scheme). All the parental strains of these two groups of plasmids were isolated at various times in different hospital locations validating the clonal dissemination of these two types of ESBL-encoding strain. Conversely, we observed the dissemination of single plasmids. Indeed, in sub-branch Ic, three closely related *bla*_CTX-M-14_ plasmids (RCS101, RCS62 and RCS68) (Fig. 6a), were found in strains of three different phylogenetic backgrounds (phylogroup A, D and B2, respectively). Thus, we did not evidence any predominant association between branches/sub-branches of the tree and ESBL type.

### IncI1 plasmids of cluster 2

In contrast to the IncF plasmids, the major part of the backbones of the IncI1 plasmids are highly conserved [63]. Thus, we were able to build a tree with 52 shared genes that included all the 17 IncI1 plasmids and excluded the IncB/O and IncK plasmids. The phylogenetic tree obtained showed that the plasmids were distributed in four main branches, I to IV (Fig. 6b). pMLST assigned the plasmids to 10 different pSTs. The correlation between the pSTs and the distribution of the plasmids in the branches of the tree was consistent as all the plasmids assigned to the same pST were found in the same branch. However, as previously observed [63], clusters of plasmids of different pSTs were found in a same sub-branch stressing a close evolutionary relationship between the different pST lineages. This was the case for plasmids of pST7 and pST3 clustered in sub-branch IIb and plasmids of pST31 and pST36 clustered in sub-branch IIIb. The two plasmids isolated before the use of the 3GCs were found clustered in two branches of the phylogenetic tree, branches II and III, along with the ESBL-encoding plasmids showing as for the IncF plasmids that the IncI1 ESBL-encoding plasmids have the same diversity and phylogenetic history as the plasmids isolated before the use of the 3GCs.

Various ESBL-encoding genes were found on the plasmids distributed in the two main branches of the tree (*bla*_SHV-12_, *bla*_CTX-M-1_ and *bla*_CTX-M-2_ gene in branch II, and *bla*_TEM-52_, *bla*_CTX-M-1_ and *bla*_CTX-M-15_ gene in branch III) and *bla*_CTX-M-1_ gene, the most represented ESBL-encoding gene among the IncI1 plasmids of this study, was found on distantly related plasmids distributed in three branches, II, II and IV. However, IncI1 plasmids that closely clustered together in sub-branches were of the same pST type and carried the same ESBL-encoding gene. Then, in sub-branch IIIb, all the plasmids of pST36 carried a *bla*_TEM-52_ gene and all the plasmids of pST31 carried a *bla*_CTX-M-15_ gene inserted in a Tn2-*bla*_TEM-1_ transposon, and in branch IIb all the plasmids of pST3 carried a *bla*_CTX-M-1_ gene with the same accessory resistance module (Fig. 6b). This was in agreement with previous works showing circulation of a number of prevalent IncI1 plasmids among bacterial species of animal and human reservoirs [63–66].

The background of the parental strains was diverse in term of phylogenetic group and MLST IP, which validated the clonal dissemination of the plasmids of the three pST-ESBL combination groups cited above. Only the two plasmids of pST232 (RCS48_pI and RCS49_pI) in branch I originated from strains of the same phylogenetic background (B2-ST9). These plasmids, which co-transferred by conjugation MOB_RNA_ plasmids harbouring *bla*_SHV-I2_, were isolated at different times in different hospital locations, suggesting in this case the dissemination of a clonal SHV-12-producing strain.

### *bla*_CTXM-15_ insertion site environment

**Fig. 7.**
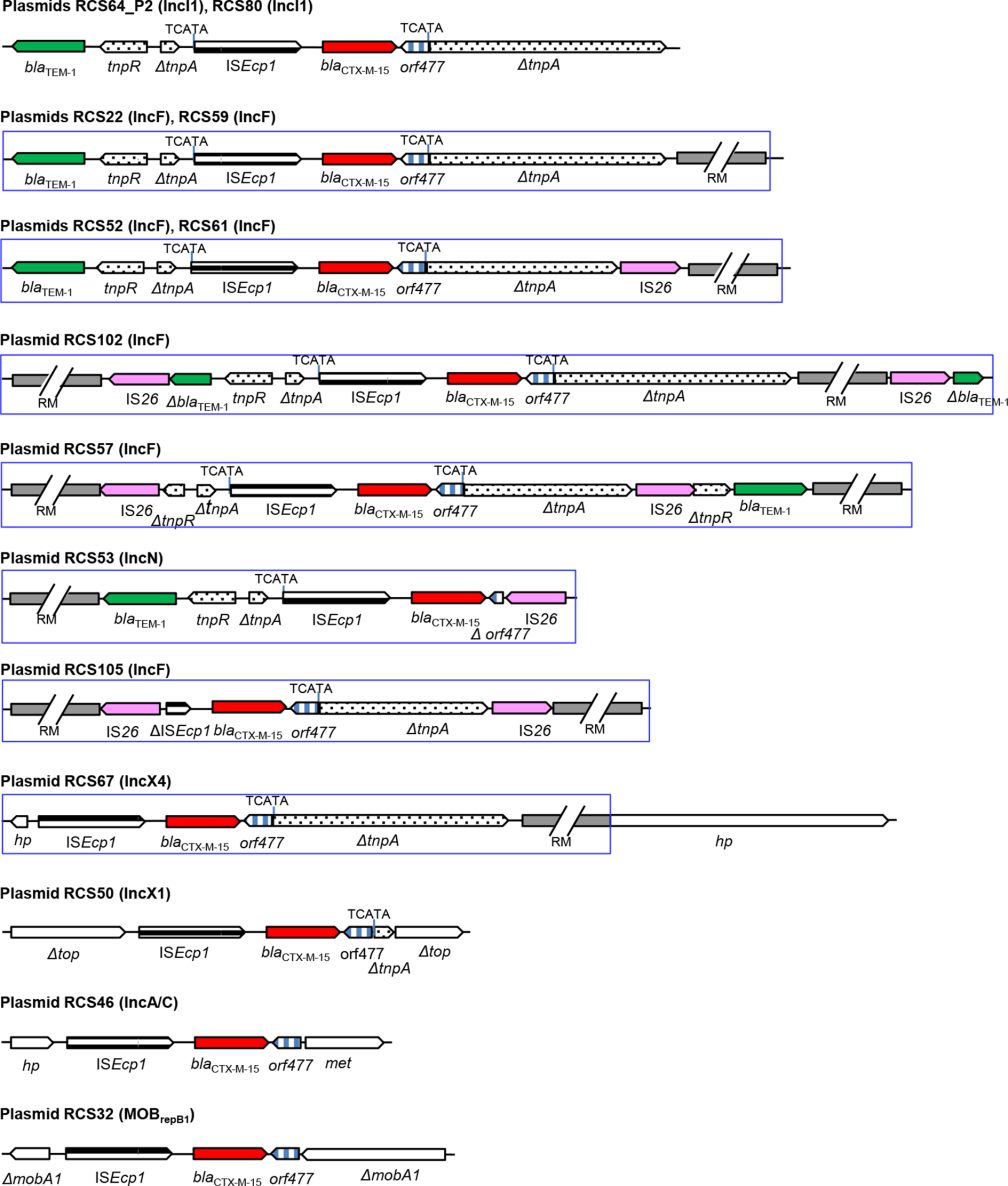
Schematic representation of the genetic environment of *bla*_CTX-M-15_ genes found in 14 plasmids of the study. The transposition unit IS*Ecp1-bla*_CTX-M-15_-*orf477* was inserted in 12 plasmids at a specific target duplicate site (TCATA) in *tnpA* of a *bla*_TEM-1_-Tn*2* transposon. *tnpA*, transposase of Tn*2* transposon; *tnpR,* resolvase of Tn*2* transposon; *top*, topoisomerase gene; *hp*, gene encoding a hypothetical protein of unknown function*; met,* gene encoding a methylase; *mobA1,* mobilization (relaxase) gene. RM, resistance module associated with *bla*_CTX-M-15_ containing different resistance genes or identical genes with different organizations on MRRs. MRRs are framed in blue.

We explored in detail the insertion site environment of the 14 plasmids of our collection carrying *bla*_CTX-M-15_ and performed additional epidemiological analysis. We found that on 12 of the 14 plasmids (7 IncF, 2 IncI1, 1 IncN, 1 IncX1 and 1 IncX4 plasmids), the *bla*_CTX-M-15_ transposition (IS*Ecp1-bla*_CTX-M-15_-*orf477*) had happened first at the target duplicate site (TCATA) of a Tn*2* transposon (*bla*_TEM-1_-*tnpR*-Δ*tnpA*-Tn2-IS*Ecp1-bla*_CTX-M-15_-*orf477*-Δ*tnpA*) (Fig. 7). On nine plasmids, the insertion of the *bla*_CTX-M-15_ gene in the Tn*2* was found on MRRs and six times the transposition unit was truncated at various positions (IR*_tnp_* and IR_TEM_ end of Tn*2*, *orf477*, and IS*Ecp1*) by IS*26* elements (Fig. 7) putatively mediating their rearrangement on the MRRs [60]. On two plasmids, RCS50 (IncX1) and RCS67 (IncX4), the *bla*_CTX-M-15_ transposition unit was inserted at a different site but a scar of a previous transposition at the Tn*2* specific target duplicate site was evident (Fig. 7). Other ESBLs of the CTX-M-1 group, such as CTX-M-1 and CTX-M-3, have the same transposition units as CTX-M-15 with the difference that beyond the right-hand inverted repeat (IR_R_) of IS*Ecp1* is generally found, in addition to the 48 bp sequence present for the *bla*_CTX-M-15_, a 32 bp sequence for *bla*_CTX-M-1_ and a 79 bp sequence for *bla*_CTX-M-3_ [67]. In our study, none of the 11 *bla*_CTX-M-1_ genes and none of the 3 *bla*_CTX-M-3_ genes was inserted in a Tn*2* transposon (data not shown). We, thus, assessed the prevalence of the insertion of the transposition unit in Tn*2* of *bla* genes of the CTX-M-1 group carried by non-redundant strains of published and personal collections [16, 23, 68] using primers designed to overlap the insertion site upstream the IS*Ecp1* and upstream the *orf477* gene (Table S3). We tested 22 strains carrying a *bla*_CTX-M-1_ and 2 strains carrying a *bla*_CTX-M-3_ that were all negative. Of the 45 strains carrying a *bla*_CTX-M-15_, 39 (86.6 %) strains were positive (10 were positive for both PCR and 29 were positive for the PCR overlapping the insertion site upstream of the *orf477* gene).

## Discussion

To have a full picture of the spread of ESBL-encoding genes in *E. coli*, we undertook a comprehensive comparative analysis of the sequences of a large number of diverse plasmids from human and animal strains isolated over a large period of time spanning before and after the use of 3GCs. For all the plasmids, we obtained high-quality circular sequences whose annotation was manually checked.

The relationship between the type of plasmid and the type of antibiotic resistance showed that although ESBL-encoding genes are mainly found on MPF plasmids, the MOB and RelN plasmids as well as the P1-like bacteriophages also play a role, albeit modest, in the diffusion of the ESBLs, and that the small RelN plasmids play a role in the diffusion of non-ESBL resistance genes compared to the MOB plasmids.

Classification and reconstruction of the evolutionary relationship of plasmids is challenging. The great variability in their gene content, mostly due to HGT events, often blurs any phylogenetic signal. Thus, we used a gene-sharing network method that allows circumventing the noisy HGT effect on the phylogenetic history reconstruction. With the exception of the small MOB_RNA_ plasmids of clusters 11 and 13, where large HGT hindered the phylogenetic signal of the different MOB_RNA_ families, all the other plasmids clustered according to the type of their genome backbone, e.g. Inc group for MPF and the various plasmid families as described earlier for the MOB and RelN_RNA_ plasmids. Thus, the plasmids having the same type of genome backbone were linked in the same cluster independently of their type of antibiotic resistance (no resistance gene, non-ESBL resistance genes or type of ESBL-encoding genes) and consequently independently of their origin (human or animal) or their period of isolation (before or after the use of the 3GCs). For each cluster of plasmids, we checked for the diversity of the parental strains as a link between specific clones and/or phylogroups and plasmids, including ESBL-producing plasmids [1, 9, 11, 23]. Globally, we did not retrieve any association between the type of plasmids and the phylogeny of the parental strains.

The analysis at a finer scale of the evolutionary history of the plasmids for the two major clusters, the IncF and IncI1 clusters, confirmed that the ESBL-encoding plasmids have the same phylogenetic history as plasmids isolated before the use of the 3GCs, suggesting that ESBL arrived at random on pre-existing plasmids and not on particular selected plasmids. It showed that the acquisition of the various ESBL-encoding genes has happened independently through multiple events on related or unrelated plasmids and that movement of the association resistance module-*bla*_CTX-M-15_ gene had happened on the IncF plasmids. We observed also that in contrary to the IncF plasmids, the occurrence of clonal diffusion of ESBL IncI1 plasmids is important [63–66].

Among CTX-M enzymes, CTX-M-15 belonging to the CTX-M-1 group appears to be the most widespread, particularly in *E. coli* [5], and surveys in several different countries have indicated that *bla*_CTX-M-15_ is often carried on IncF plasmids [3, 11, 12, 60, 62, 69–71]. *bla*_CTX-M-15_ is found as part of a transposition unit that has been described many times, inserted into a specific target duplicate site (TCATA) in the *tnpA* of a *bla*_TEM-1_-Tn*2* transposon [60, 72, 73]. Only once, on plasmid pSH4469, has a different target duplicate site been found on a Tn*2* transposon [72]. In our study, the majority (12/14) of the *bla*_CTX-M-15_ was inserted into this specific target unlike the *bla*_CTX-M-1_ and *bla*_CTX-M-3_ genes, although they belong to the same CTX-M-1 group. However, to our knowledge, the insertion in the *tnpA* of Tn*2* had been described for *bla*_CTX-M-3_ at a different target duplicate site as on pEK204 [72, 74, 75], but has never been described for *bla*_CTX-M-1_. Our additional epidemiological analysis on strains of our collections carrying *bla* genes of the CTX-M-1 group showed that the majority of the *bla*_CTX-M-15_ genes were found inserted into the specific target, in agreement with previous observations that *bla*_CTX-M-15_ but not *bla*_CTX-M-1_ genes easily transpose on plasmids carrying *bla*_TEM-1_-Tn*2* at the specific duplicated site TCATA. In *E. coli*, ampicillin resistance is mainly conferred by the *bla*_TEM-1_ gene that is located on a Tn*2* transposon [12, 76, 77]. Ampicillin resistance in *E. coli* rises from 27 % in the community to 55 % in hospital infections, and has increased during the last two decades [76, 78–81]. Therefore, the wide spread of the CTX-M-15 enzymes, but not the CTX-M-1 nor the CTX-M-3 enzymes, among *E. coli* strains seemed to be linked to a hot spot of insertion on the *bla*_TEM-1_-Tn*2* transposon, a transposon largely found on IncF plasmids, one of the most prevalent plasmids in *E. coli* strains [12, 57, 82].

The main conclusions of our work are that ESBL-encoding genes arrived multiple times on a wide range of pre-existing plasmids and that a highly dynamic pattern of mobility was observed concerning different nested physical units represented by the ESBL-encoding gene, the MRR and finally the plasmid. The combination of these different levels multiplies the potential of ESBL-encoding gene spread. Furthermore, the successful spread of the CTX-M-15 ESBL-encoding gene in *E. coli* seemed to be favoured by its arrival in a Tn*2- bla*_TEM-1_ transposon borne on well-adapted IncF plasmids.

## Data bibliography

1. Genoscope CEA. European Nucleotide Archive, PRJEB24625 (2018).
2. Genoscope CEA. European Nucleotide Archive, LO017736 (2016).
3. Genoscope CEA European Nucleotide Archive, LO017737 (2016).
4. Genoscope CEA. European Nucleotide Archive, LO017738 (2016).
5. Genoscope CEA. European Nucleotide Archive, FO818745 (2015).
6. Genoscope CEA. European Nucleotide Archive, FQ482074 (2010).

## Supplementary Data

Supplementary File 1
