# Supplementary material for: Extended-spectrum β-lactamase-encoding genes are spreading on a wide range of Escherichia coli plasmids existing prior to the use of third-generation cephalosporins
Source: Microb Genom. 2018 Aug 6;4(9):e000203. doi: 10.1099/mgen.0.000203 (PMC6202452; doi:10.1099/mgen.0.000203)
Supplement: Supplementary File 1 [file mgen-4-203-s001.pdf]

Table S1. Characteristics of the 73 ESBL producing-strains and of the 87 plasmids studied

Parental strains								Plasmid tranfer[#]	Plasmids in the recipient strains					Reference[&]	GenBank acession no
**Strain ID**	**Host**	**Geograpic origin**	**Samples[*]**	**Year of isolation**	**Phylo group[+]**	**cMLST IP[‡]**	**ESBL production[§]**		**Plasmid ID**	**Length bp**	**Inc group[¶]**	**pMLST**	**ESBL gene**		
03-235	*Gallus gallus*	France	Bacteraemia	2003	D	48	+	C	RCS72	108.661	I1	ST3	$bla_{\text{CTX-M-1}}$	(1)	LT985273
03-237	*Sus scrofa domestica*	France	UTI	2004	D	597	+	C	RCS73	93.239	I1	ST49	$bla_{\text{CTX-M-1}}$	(1)	LT985288
13942	*Bos taurus*	France	Diarrhea	2004	NA		+	C	RCS74 _pI	94.836	I1	ST179	$bla_{\text{CTX-M-1}}$	(1)	LT985283
									RCS74_pII	7.133	MOB_{RNA}		-		LT985282
13947	*Gallus gallus*	France	Bacteraemia	2004	A	478	+	C	RCS75_pI	108.379	I1	ST3	$bla_{\text{CTX-M-1}}$	(1)	LT985280
13948	*Bos taurus*	France	UTI	2004	C	7	+	C	RCS76_pI	110.066	I1	ST3	$bla_{\text{CTX-M-1}}$	(1)	LT985286
									RCS76_pII	6.549	MOB_{RNA}		-		LT985284
									RCS76_pIII	4.054	$\text{MOB}_{\text{repB1}}$		-		LT985285
1454	*Equus caballus*	Belgium	Funiculitis	2009	A	2	+	C	RCS78	239.460	HI1, FIA, Q-1	ST9	$bla_{\text{CTX-M-2}}$	(3)	LT985296
4410	*Equus caballus*	Belgium	Arthritis	2007	A	736	+	C	RCS79	168.237	FII, FIB, Q-1	F18:A-:B1	$bla_{\text{CTX-M-2}}$	(3)	LT985293
B1-54	*Gallus gallus*	Belgium	Faeces	2007	A	715	+	C	RCS71	91.635	I1	ST36	$bla_{\text{TEM-52c}}$	(2)	LT985281
B4-25	*Gallus gallus*	Belgium	Faeces	2007	D	45	+	C	RCS77	235.943	HI2	ST2	$bla_{\text{CTX-M-2}}$	(2)	LT985297
B4-75	*Gallus gallus*	Belgium	Faeces	2007	B1	88	+	C	RCS80	87.487	I1	ST31	$bla_{\text{CTX-M-15}}$	(2)	LT985289
83	*Homo sapiens*	France	Faeces	1998	A	716	+	C	RCS1TR83	174,073	A/C		$bla_{\text{TEM-24}}$	(4) (5)	LT985220
89	*Homo sapiens*	France	Faeces	1999	C	717	+	C	RCS2TR89	175,317	A/C		$bla_{\text{TEM-24}}$	(4) (5)	LT985225
167	*Homo sapiens*	France	UTI	1998	B1	88	+	E	RCS39	85.621	A/C		$bla_{\text{CTX-M-2}}$	(4) (5)	LT985244
170	*Homo sapiens*	France	Misc. infection	1998	A	2	+	C	RCS38	211.452	A/C, N2		$bla_{\text{SHV-12}}$	(4) (5)	LT985250
177	*Homo sapiens*	France	Misc. colonisation	1998	A	664	+	C	RCS53	54.684	N	ST6	$bla_{\text{CTX-M-15}}$	(4) (5)	LT985266
195	*Homo sapiens*	France	Faeces	1999	A	718	+	C	RCS46	216.620	A/C, R		$bla_{\text{CTX-M-15}}$	(4) (5)	LT985249
364	*Homo sapiens*	France	Faeces	2002	B2	27	+	C	RCS57TR364	98.763	FII	F2:A-:B-	$bla_{\text{SHV-12}}$	(4) (5)	LT985265
473	*Homo sapiens*	France	Misc. infection	2001	D	737	+	E	RCS52	134.392	FII, FIA, FIB	F22:A4:B1	$bla_{\text{CTX-M-15}}$	(4) (5)	LO017736
497	*Homo sapiens*	France	UTI	2001	B2	4	+	E	RCS21	121.741	FII, FIB	F51:A-:B10	$bla_{\text{SHV-2 (2)}}$	(4) (5)	LT985219
499	*Homo sapiens*	France	UTI	2000	D	719	+	C	RCS68	86.943	K		$bla_{\text{CTX-M-14}}$	(4) (5)	LT985278
502	*Homo sapiens*	France	UTI	2000	C	74	+	E	RCS45	41.540	N	ST1	$bla_{\text{CTX-M-1}}$	(4) (5)	LT985251
TN03	*Homo sapiens*	France	UTI	2001	B2	43	+	E	RCS22	128.281	FII, FIA	F2:A1:B-	$bla_{\text{CTX-M-15}}$	(6)	LT985221
506	*Homo sapiens*	France	UTI	2001	D	720	+	E	RCS62_pI	70.033	FII	F2:A-:B-	$bla_{\text{CTX-M-14}}$	(4) (5)	LT985272
511	*Homo sapiens*	France	Faeces	1994	B1	88	+	E	RCS54	137.900	P1-like phage, Q-1		$\text{bla}_{\text{CTX-M-9}}$	(4) (5)	LT985263
513	*Homo sapiens*	France	Bacteraemia	1999	F	721	+	C	RCS30	157.836	A/C		$bla_{\text{CTX-M-14}}$	(4) (5)	LT985224
518	*Homo sapiens*	France	UTI	2001	B1	349	+	E	RCS28	95.442	FII	F2:A-:B-	$bla_{\text{SHV-12}}$	(4) (5)	LT985227
522	*Homo sapiens*	France	UTI	1995	A	2	+	C	RCS65	121.498	FII	F2:A-:B-	$bla_{\text{CTX-M-14}}$	(4) (5)	LT985277
523	*Homo sapiens*	France	UTI	1997	D	722	+	C	RCS42	42.311	N	ST1	$bla_{\text{CTX-M-1}}$	(4) (5)	LT985245
525	*Homo sapiens*	France	UTI	1998	B2	4	+	C	RCS27	118.656	FIB, FII	F51:B10	$bla_{\text{SHV-2}}$	(4) (5)	LT985226
533	*Homo sapiens*	France	UTI	1999	B2	33	+	E	RCS12TR533	14.248	MOB_{RNA}		$bla_{\text{TEM-3}}$	(4) (5)	LT985216
541	*Homo sapiens*	France	Faeces	2000	A	723	+	E	RCS18TR541	130.621	A/C		$bla_{\text{TEM-21}}$	(4) (5)	LT985218
548	*Homo sapiens*	France	Misc. infection	2000	A	2	+	E	RCS24TR548	175.038	A/C		$bla_{\text{TEM-24}}$	(4) (5)	LT985222
552	*Homo sapiens*	France	UTI	2001	A	2	+	E	RCS28TR552	157.534	A/C		$bla_{\text{TEM-3}}$	(4) (5)	LT985228
557	*Homo sapiens*	France	Faeces	2001	A	11	+	E	RCS32TR557	93,070	Y		$bla_{\text{TEM-24}}$	(4) (5)	LT985234
565	*Homo sapiens*	France	Bacteraemia	2002	B1	87	+	E	RCS35_pI	4.087	$\text{MOB}_{\text{repB1}}$		-	(4) (5)	LT985232
									RCS35_pII	14.365	MOB_{RNA}		$bla_{\text{SHV-12}}$		LT985233
580	*Homo sapiens*	France	Bacteraemia	2002	B2	9	+	C	RCS49_pI	80.790	I1	232	-	(4) (5)	LT985256
									RCS49_pII	4.074	$\text{MOB}_{\text{repB1}}$		-		LT985255
									RCS49_pIII	15.843	MOB_{RNA}		$bla_{\text{SHV-12}}$		LT985254
									RCS49_pIV	20.798	MOB_{RNA}		$bla_{\text{SHV-12}}$		LT985257

592	*Homo sapiens*	France	UTI	2002	B1	632	+	C	RCS44	42.084	N	ST1	$bla_{\text{CTX-M-1}}$	(4) (5)	LT985259
604	*Homo sapiens*	France	UTI	2002	C	738	+	E	RCS43	44,494	N	ST1	$bla_{\text{CTX-M-1}}$	(4) (5)	LT985242
631	*Homo sapiens*	France	Faeces	2002	A	2	+	E	RCS63	22.308	$RelN_{RNA}$		$bla_{\text{CTX-M-14}}$	(4) (5)	LT985269
637	*Homo sapiens*	France	UTI	2002	F	724	+	C	RCS61	87.290	FII	F2:A-:B-	$bla_{\text{CTX-M-15}}$	(4) (5)	LT985267
641	*Homo sapiens*	France	Faeces	2002	B1	739	+	C	RCS33_pI	88.922	I1	STI36	$bla_{\text{TEM-52c}}$	(4) (5)	LT985236
									RCS33_pII	6.647	MOB_{RNA}		-		LT985237
649	*Homo sapiens*	France	Faeces	2002	A	2	+	C	RCS101	70,309	FII	F2:A-:B-	$bla_{\text{CTX-M-14}}$	(4) (5)	LT985214
650	*Homo sapiens*	France	Faeces	2002	D	740	+	C	RCS64_pI	3,002	MOB_{RNA}		-	(4) (5)	LT985294
									RCS64_pII	91,307	I1	ST31	$bla_{\text{CTX-M-15}}$		LT985295
654	*Homo sapiens*	France	UTI	2002	A	2	+	C	RCS34	111.333	I1	ST3	$bla_{\text{CTX-M-1}}$	(4) (5)	LT985235
657	*Homo sapiens*	France	UTI	2000	A	664	+	E	RCS50	42.913	X1		$bla_{\text{CTX-M-15}}$	(4) (5)	LT985261
659	*Homo sapiens*	France	UTI	2001	B2	43	+	E	RCS32	7.050	MOB_{repB1}		$bla_{\text{CTX-M-15}}$	(4) (5)	LT985240
660	*Homo sapiens*	France	Faeces	2001	A	398	+	C	RCS48TR660	157,228	A/C		$bla_{\text{TEM-52}}$	(4) (5)	LT985253
661	*Homo sapiens*	France	UTI	2002	B2	43	+	C	RCS59	130.559	FII, FIA	F2:A1:B-	$bla_{\text{CTX-M-15}}$	(4) (5)	LT985271
666	*Homo sapiens*	France	UTI	2002	D	8	+	E	RCS51	179,021	FII, FIB	F18:A-:B34	$bla_{\text{CTX-M-9}}$	(4) (5)	LT985252
676	*Homo sapiens*	France	Bacteraemia	2002	B2	43	+	E	RCS60	63.009	L/M		$bla_{\text{CTX-M-14}}$	(4) (5)	LT985279
690	*Homo sapiens*	France	UTI	2002	A	741	+	E	pRCS57	143.225	FII, FIA, FIB	F31:A4:B1	$bla_{\text{CTX-M-15}}$	(4) (5)	LO017738
692	*Homo sapiens*	France	UTI	2001	C	74	+	E	RCS29_pI	89.006	I1	ST36	$bla_{\text{TEM-52c}}$	(4) (5)	LT985238
									RCS29_pII	33.899	X-like		-		LT985239
694	*Homo sapiens*	France	UTI	2001	A	2	+	C	RCS55_pI	90.270	L/M		$bla_{\text{CTX-M-3}}$	(4) (5)	LT985387
									RCS55_pII	6,859	MOB_{RNA}		-		LT985386
699	*Homo sapiens*	France	UTI	2002	B1	24	+	C	RCS58	117.655	I1	ST29	$bla_{\text{CTX-M-2}}$	(4) (5)	LT985268
707	*Homo sapiens*	France	UTI	2002	B2	43	+	C	RCS67	63.787	X4		$bla_{\text{CTX-M-15}}$	(4) (5)	LT985276
708	*Homo sapiens*	France	UTI	2002	B2	43	+	C	RCS102	129.394	FII, FIA	F2:A1:B-	$bla_{\text{CTX-M-15}}$	(4) (5)	LT985213
711	*Homo sapiens*	France	Faeces	2002	B2	10	+	E	RCS25	42.584	N2		$bla_{\text{SHV-5}}$	(4) (5)	LT985223
713	*Homo sapiens*	France	UTI	1989	B1	742	+	C	RCS36	217,183	FIIK, FIA	FK7:A10:B-	$bla_{\text{SHV-3}}$	(4) (5)	LT985229
715	*Homo sapiens*	France	UTI	2003	D	8	+	E	RCS103	67.968	FII	F4:A-:B-	$bla_{\text{CTX-M-14}}$	(4) (5)	LT985217
716	*Homo sapiens*	France	UTI	2002	B2	221	+	E	RCS56	90.206	K		$bla_{\text{CTX-M-14}}$	(4) (5)	LT985270
717	*Homo sapiens*	France	Faeces	2003	A	716	+	C	RCS51TR717	86,770	L/M		$bla_{\text{TEM-3}}$	(4) (5)	LT985264
719	*Homo sapiens*	France	Faeces	2002	B2	743	+	E	RCS52TR719	38.610	X1		$bla_{\text{TEM-52}}$	(4) (5)	LT985262
720	*Homo sapiens*	France	UTI	2003	B2	9	+	C	RCS48_pI	80.596	I1	232	-	(4) (5)	LT985248
									RCS48_pII	19.813	MOB_{RNA}		$bla_{\text{SHV-12}}$		LT985247
									RCS48_pIII	4.772	MOB_{RNA}		-		LT985246
721	*Homo sapiens*	France	Faeces	2003	B2	725	+	E	RCS40	90.291	L/M		$bla_{\text{CTX-M-3}}$	(4) (5)	LT985241
722	*Homo sapiens*	France	Faeces	2002	B2	36	+	C	RCS41	159.550	A/C		$bla_{\text{TEM-3}}$	(4) (5)	LT985243
723	*Homo sapiens*	France	Misc. infection	2002	B2	506	+	C	RCS66	70.325	FII	F2:A-:B-	$bla_{\text{CTX-M-14}}$	(4) (5)	LT985274
724	*Homo sapiens*	France	Faeces	2002	B1	726	+	C	RCS53TR724	157.225	A/C		$bla_{\text{TEM-3}}$	(4) (5)	LT985260
725	*Homo sapiens*	France	UTI	2002	B1	744	+	E	RCS47	115.154	P1-like phage		$bla_{\text{SHV-2}}$	(4) (5)	FO818745
726	*Homo sapiens*	France	Faeces	2002	B2	745	+	C	RCS54TR726	157.227	A/C		$bla_{\text{TEM-21}}$	(4) (5)	LT985258
727	*Homo sapiens*	France	Faeces	2003	B2	4	+	E	RCS55TR727	40.984	RelN IncR		$bla_{\text{TEM-52}}$	(4) (5)	LT985322
730	*Homo sapiens*	France	UTI	2002	F	IC	+	C	RCS37_pI	10.551	MOB_{RNA}		-	(4) (5)	LT985230
									RCS37_pII	114.286	I1	ST178	$bla_{\text{SHV-12}}$		LT985231
O102-ST405 (TN48)	*Homo sapiens*	France	UTI	2004	D	727	+	E	ETN48	165.657	FII, FIB, Q-1	F22:A-:B1	$bla_{\text{CTX-M-14}}$	(7)	FQ482074
3249 (RD29512)	*Homo sapiens*	France	Misc. colonisation	2009	A	2	+	E	RCS105	175.264	FII, FIA, FIB	F31:A4:B1	$bla_{\text{CTX-M-15}}$	(8)	LO017737

[*] UTI, urinary tract infection; [+]NA , non available; [‡]IC, imcomplete sequence type; [§]+, positive; -, negative; [#]C, conjugation; E, electroporation

[¶] MOBRep, mobilizable plasmid with a replication protein system; MOBRNA,mobilizable plasmid with a RNAII/RNAI replication/control system; RelN, relaxase negative plasmid; RelNRNA relaxase negative plasmid with a RNAII/RNAI replication/control system.

[&] References, (1) Meunier D et al. (2006) Int J Antimicrob Agents 28:402-407; (2) Smet A et al. (2009) J Antimicrob Chemother 63:1286-1288; (3) Smet A et al. (2012) J Antimicrob Chemother 67:1289-1291; (4) Branger C et al. (2005) Emerg Infect Dis 11:54-61; (5) Marcade G et al. (2009) J Antimicrob Chemother 63:67-71; (6) Lavollay M et al. (2006) Antimicrob Agents Chemother 50:2433-2438; (7) Deschamps C et al.(2009) Microbiology 155:1656-1668; (8) Birgy A et al.(2013) J Clin Microbiol 51:1727-1732.

Table S2. Characteristics of the 19 strains isolated before the use of the 3GC and of the29 plasmids studied

Parental strains								Plasmid tranfer[#]	Plasmids of recipient strains					Reference[&]	GenBank accession no
Strain identity	Host	Geograpic origin	Samples[*]	Year of isolation	Phylo group+	cMLST[+] IP	antibiotype		Plasmid identity	length	Inc group[¶]	pMLST[‡]	Resistance genes[§]		
ECOR 3	*Canis lupus*	USA, Ma	faeces	70s-80s	A	182	Str Tet Sul	C	RCS85_pI	117.804	I1	IC	*aadA1 tetA*(A) *sul1*	(1)	LT985321
									RCS85_pII	102.718	B/O		-		LT985320
ECOR 4	*Homo sapiens*	USA, IA	faeces	70s-80s	A	188	Str Tet Sul	E	RCS86_pI	6.069	RelN$_{RNA}$		*sul2*	(1)	LT985307
ECOR 6	*Homo sapiens*	USA, IA	faeces	70s-80s	A	IC	Str Tet	E	RCS91	13.593	MOB$_{RNA}$		*tetA*(A)	(1)	LT985299
ECOR 10	*Homo sapiens*	USA, NY	faeces	70s-80s	A	2	Sensitive	None	RCS83_pI	4.074	RelN$_{RNA}$		-	(1)	LT985290
									RCS83_pII	2.999	RelN$_{RNA}$		-		LT985291
									RCS83_pIII	5.912	MOB$_{RNA}$		-		LT985292
ECOR 17	*Sus scrofa domestica*	Indonesia	faeces	70s-80s	A	169	Str Tet Sul	E	RCS98	6.200	RelN$_{RNA}$		*strA/B sul2*	(1)	LT985316
ECOR 18	*Macaca nigra*	USA, WA	faeces	70s-80s	A	132	Sul	E	RCS87	6.047	RelN$_{RNA}$		*sul2*	(1)	LT985298
ECOR 19	*Macaca nigra*	USA, WA	faeces	70s-80s	A	170	Str Tet Sul	E	RCS81	37.543	X2		*tetA*(A)	(1)	LT985317
ECOR 24	*Homo sapiens*	Sweden	faeces	70s-80s	A	176	Str Sul	C	RCS90_pI	92.968	B/O		*strA/B sul2*	(1)	LT985300
									RCS90_pII	11.964	MOB$_{repB2}$		*strA/B*		LT985301
ECOR 25	*Canis lupus familiari*	USA, NY	faeces	70s-80s	A	177	Str Tet Sul	E	RCS92_pI	6.222	RelN$_{RNA}$		*strA/B sul2*	(1)	LT985314
									RCS92_pIII	3.174	RelN$_{RNA}$		-		LT985312
ECOR 30	*Bison bison*	Canada, AB	faeces	70s-80s	B1	183	Str Tet	C	RCS96_pI	37.454	X 4		-	(1)	LT985318
									RCS96_pII	147.478	FII, FIB	F24:A-:B1	*strA/B tetA*(B)		LT985319
ECOR 31	*Panthera pardus*	USA, WA	faeces	70s-80s	E	250	Amp Str kan Tet Sul	C	RCS89	89.661	FII	F2:A-:B-	bla_{TEM-1} *aadA1 aphA-1b tetA*(B) *sul1*	(1)	LT985304
ECOR 37	*Callithrix*	USA, WA	faeces	70s-80s	E	187	Str kan Tet Sul Cm	E	RCS88	93.395	FII	F2:A-:B-	*aadA1 aphA-1 tetA*(A) *sul1 cat*	(1)	LT985306
ECOR 39	*Homo sapiens*	Sweden	faeces	70s-80s	F	254	Sensitive	None	RCS82_pI	47.789	Phage P2 like		-	(1)	LT985302
									RCS82_pII	23.526	MOB$_{RNA,}$ FIB		-		LT985303
ECOR 48	*Homo sapiens*	Sweden	UTI	70s-80s	D	256	Str Tet Sul	C	RCS84	83.309	FII	F2:A-:B-	*aadA1 tetA(C) sul1*	(1)	LT985305
ECOR 70	*Gorilla gorilla*	USA, WA	faeces	70s-80s	C	211	Sul	C	RCS99	47.447	X1		*sul2*	(1)	LT985315
IAI 27	*Homo sapiens*	France	UTI	80s	D	IC	Cm	E	RCS100	106.000	I1	ST7	*cat*	(2)	LT985215
R71	*Homo sapiens*	France	faeces	1969	NA		Amp Str Tet Sul Cm	C	RCS70	91.487	FII	F6:A-:B-	*blaSHV-1 aadAI tetA*(D) *cat sul1*	(3)	LT985275
RPC3	*Homo sapiens*	Canada	faeces	1958	NA		Str Kan	C	RCS69_pII	49.251	N-like		*strA/B aphA-2*	(3)	LT985287
CIP106223	*Homo sapiens*	UK	NA	1932	F	IC	Sensitive	None	RCS93_pI	83.523	FII, FIB	F29:A-:B1	-	(4)	LT985309
									RCS93_pII	35.258	IncUN		-		LT985310
									RCS93_pIII	5.631	MOB$_{RNA}$		-		LT985311
									RCS93_pIV	4.913	MOB$_{RNA}$		-		LT985308

[*] UTI, urinary tract infection; [+]NA , non available; [‡]IC, imcomplete sequence type;; [#]C, conjugation; E, electroporation ; § -, absence

[¶] MOBRep, mobilizable plasmid with a replication protein system; MOBRNA,mobilizable plasmid with a RNAII/RNAI replication/control system; RelN, relaxase negative plasmid; RelNRNA relaxase negative plasmid with a RNAII/RNAI replication/control system; UN unknown.

[&] References, (1) Ochman H and Selander RK. (1984) J Bacteriol 157:690-693; (2) Picard B et al. (199 Infect Immun 67:546-553; (3)INRA, UR1282 collection; (4) Hughes VM and Datta N. (1983) Nature 302:725-726.

Table S3. Primers used to detect the insertion site of the *bla*$_{CTX-M-15}$ transposition unit in the *tnpA* of Tn*2* transposon (*bla*$_{TEM-1}$-*tnpR*-Δ*tnpA*-Tn2-IS*Ecp1*-*bla*$_{CTX-M-15}$-*orf477*-Δ*tnpA*)

PCR target	Primer name	Primer sequence *
	**Primer pair 1**	
ΔtnpA-IS*Ecp1* *region*	tnpA-ISEcp1 FW	5'-CCGATATGCCCGTTGACTT-3'
	tnpA-ISEcp1 Rev	5'- CCGCAGAGCATGGTCTTTAT-3'
	**Primer pair 2**	
orf 477-ΔtnpA region	orf 477-tnpA Fw	5'-GGCAGCCACGGGTTGATA-3'
	orf 477-tnpA Rev	5'-AAGACGGGTAAGGCGAAATG-3'

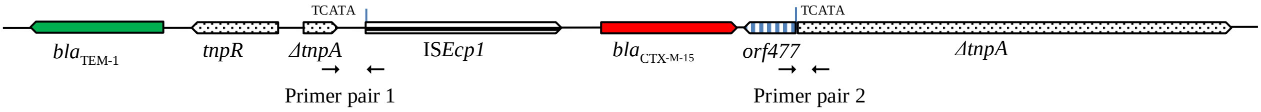

*Schematic representation of the insertion site of the ***bla***$_{CTX-M-15}$ transposition unit in the Tn*2* transposon
